# Comparison of the human-baited double net trap with the human landing catch for *Aedes albopictus* monitoring in Shanghai, China

**DOI:** 10.1186/s13071-018-3053-8

**Published:** 2018-08-28

**Authors:** Qiang Gao, Fei Wang, Xihong Lv, Hui Cao, Jianjun Zhou, Fei Su, Chenglong Xiong, Peien Leng

**Affiliations:** 1 Department of Vector & Parasite Control, Huangpu Center for Disease Control & Prevention, Shanghai, 200023 People’s Republic of China; 2 Department of Vector & Parasite Control, Hongkou Center for Disease Control & Prevention, Shanghai, 201620 People’s Republic of China; 3 Department of Vector & Parasite Control, Songjiang Center for Disease Control & Prevention, Shanghai, 200082 People’s Republic of China; 4 Department of Medical Microbiology, School of Public Health, Fudan University, Shanghai, 200032 People’s Republic of China; 5 Department of Vector & Parasite Control, Shanghai Municipal Center for Disease Control & Prevention, Shanghai, 200336 People’s Republic of China

**Keywords:** Human-baited double net trap (HDN), Human landing catch (HLC), *Aedes albopictus*, Downtown Shanghai

## Abstract

**Background:**

Human landing catch (HLC) is the most efficient method for *Aedes* monitoring, but it is not ethical due to its high risk of human exposure to pathogens. We designed trials to assess the performance of an alternative human-baited double net trap (HDN) for field *Aedes albopictus* monitoring compared with the standard HLC.

**Methods:**

Outdoor HDN and HLC catches were conducted simultaneously at 15 field sites on two sunny days in mid-July and August. The tests were performed 3 h apart: an early morning period (7:30–8:30 h), a pre-sunset period (16:30–17:30 h) and a post-sunset period (18:30–19:30 h). A total of 90 comparisons were made between the two methods. Field comparisons were designed to minimize half-hour bias and human-bait attraction bias.

**Results:**

Two mosquito species were collected by HDN and HLC, with the predominated species being *Ae. albopictus* (HDN: *n* = 1325, 97.35% of total; HLC: *n* = 531, 92.51% of total). A small proportion were adults of the *Culex pipiens* complex (HDN: *n* = 36, 2.65% of total; HLC: *n* = 43, 7.49% of total). Although the mean *Ae. albopictus* catch per hour of HLC was significantly higher than HDN (14.72 *vs* 5.90 per h, *t*_(178)_ = 3.151, *P* = 0.003), there were significant positive spatial and temporal correlations between HLC and HDN for *Ae. albopictus* sampling among different sites and hours (*r*_(90)_ = 0.785, *P* < 0.001; *r*_(90)_ = 0.785, *P* < 0.001). Both methods proved that *Ae. albopictus* was most active during the hours before sunset and least active after sunset. No significant variation was observed in *Ae. albopictus* catch size of HDN between groups of more attractive and less attractive humans (3.38 *vs* 2.51 per 30 min, *t*_(88)_ = 1.283, *P* = 0.201).

**Conclusions:**

With moderate sampling efficiency, significantly positive spatial correlation with HLC, and less human-bait attraction bias, HDN appears to be a safer alternative to HLC for *Ae. albopictus* monitoring in Shanghai. With mosquito activity peaking in the pre-sunset hours, *Ae. albopictus* catches of HDN should be performed in the hours before dark. The trap design could be improved to make it more portable and easier for field operation.

## Background

The Asian tiger mosquito, *Aedes albopictus* (Skuse), is the predominant mosquito vector species in Shanghai, China [1, 2]. *Aedes albopictus* is also the most annoying, invasive and perhaps the most dangerous vector species in Shanghai, due to its highly anthropophilic host preference and potential role in transmitting dengue, chikungunya or Zika viruses [3]. The public health threat posed by *Ae. albopictus* has made it the main focus of vector control efforts in Shanghai and surrounding areas. Monitoring adult *Ae. albopictus* is the major means of evaluating vector density, vector-borne disease risk and the efficacy of vector-control operations. A useful monitoring method combining safety and accuracy is badly needed for *Ae. albopictus* management.

For highly anthropophilic mosquitoes, none of the existing traps without human attractants are as effective as the traditional human landing catch method (HLC) [4–8]. The HLC lures host-seeking mosquitoes with a human attractant, and the mosquitoes are collected when they land on exposed arms or legs. Although the HLC is considered to be the most effective method for *Aedes* sampling because of its high sensitivity and efficiency [9], it poses the risks of humans contracting mosquito-borne pathogens, especially when mosquito-borne diseases like dengue or Zika are endemic [10]. For example, Simard et al. [11] did not use HLC for sampling *Aedes aegypti* and *Ae. albopictus*, due to the risks of contracting dengue and the lack of a vaccine or effective treatment. Hence, the city of Shanghai introduced the human-baited double net trap (HDN) as an alternative for *Aedes* monitoring.

The HDN consists of two box nets; the inner net protects the human-bait, and the outer net is raised off the ground so that mosquitoes lured to the human-bait are collected between the nets by another collector, who is protected by repellent. Compared to HLC, HDN also uses human-bait as a mosquito attractant, but the human-bait is protected from mosquito landing and biting. The outer collector is protected by long-sleeved clothing and repellent, which compensates for the unsafe shortcomings of HLC. However, no comparisons of *Aedes* sampling efficiency for the HDN method *vs* HLC have been carried out in China, and there are few related studies.

In this study, we assessed the efficiency of HDN for adult *Ae. albopictus* monitoring in Shanghai and compared this method to HLC. Our objective was to assess the advantages and disadvantages of HDN for mosquito monitoring. We also evaluated the potential of HDN to replace HLC for emergency *Ae. albopictus* monitoring and determined the catch conversion index between HDN and HLC.

## Methods

### Study area

**Fig. 1:**
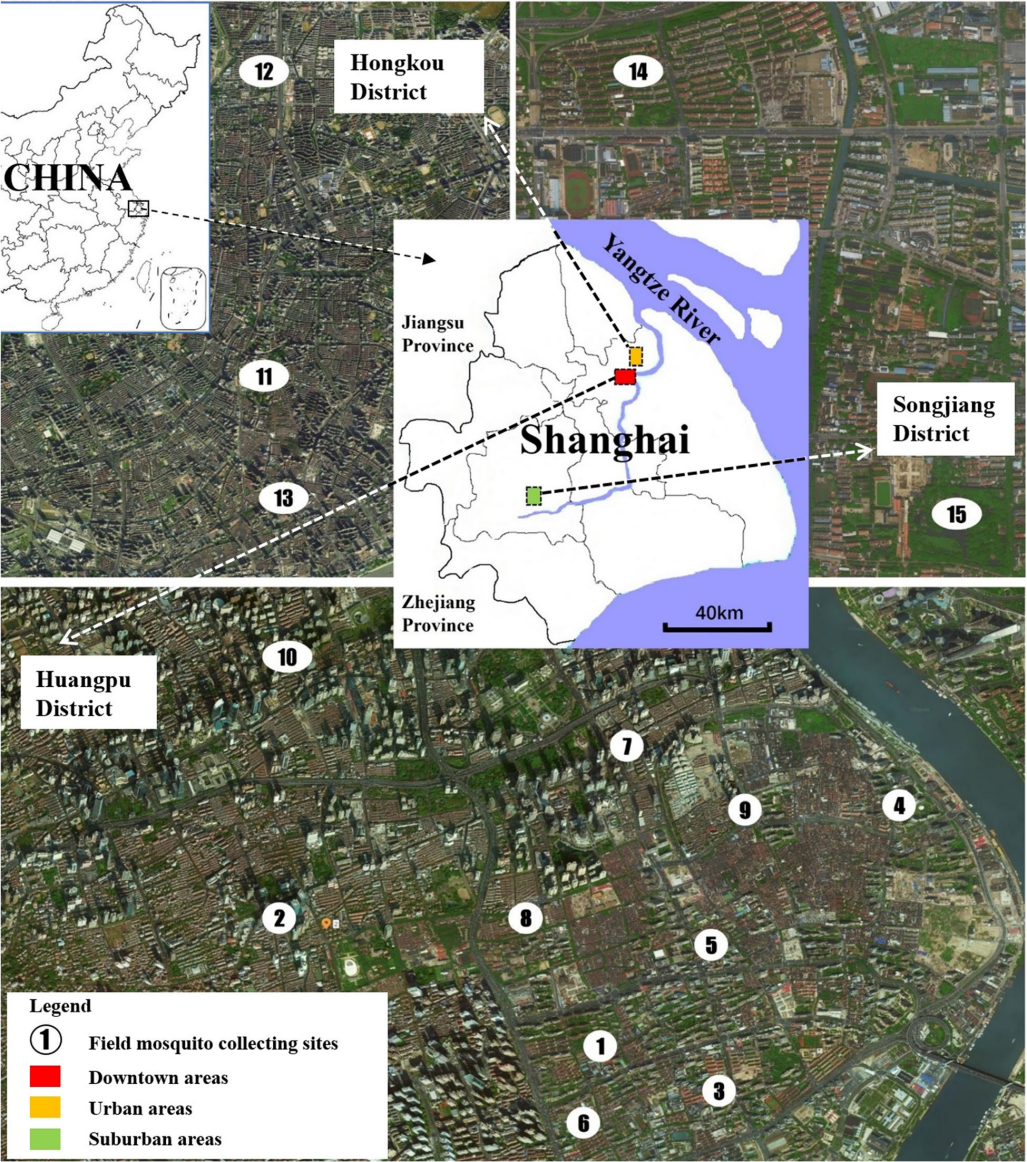
Locations of the 15 field sites for mosquito monitoring comparison between HDN *vs* HLC

The studies were conducted in the eastern China city of Shanghai. A total of 15 field monitoring sites scattered among 3 districts representing downtown, urban and suburban environments respectively, were selected for mosquito sampling. Details of location and site characteristics are shown in Fig. 1 and Table 1.

**Table 1:** Geographical information of 15 sites for mosquito monitoring comparison between HDN and HLC

Site ID	District	Type of environment	Coordinate
Site 1	HP	Enterprise and institution	31°12'30.52"N, 121°28'28.41"E
Site 2	HP	Enterprise and institution	31°12'58.66"N, 121°27'21.21"E
Site 3	HP	Residential neighborhood	31°12'21.48"N, 121°28'56.92"E
Site 4	HP	Residential neighborhood	31°13'27.99"N, 121°29'41.63"E
Site 5	HP	Residential neighborhood	31°12'55.88"N, 121°28'55.00"E
Site 6	HP	Residential neighborhood	31°12'12.97"N, 121°27'23.76"E
Site 7	HP	High school	31°13'41.97"N, 121°28'33.78"E
Site 8	HP	Enterprise and institution	31°13'00.54"N, 121°28'09.46"E
Site 9	HP	Residential neighborhood	31°13'27.14"N, 121°29'03.28"E
Site 10	HP	Residential neighborhood	31°14'09.61"N, 121°27'47.84"E
Site 11	HK	Parks or green areas	31°16'29.63"N, 121°28'44.32"E
Site 12	HK	High school	31°18'49.19"N, 121°28'40.52"E
Site 13	HK	Parks or green areas	31°15'24.64"N, 121°28'49.29"E
Site 14	SJ	Residential neighborhood	31°01'15.50"N, 121°13'45.93"E
Site 15	SJ	Parks or green areas	31°00'25.18"N, 121°14'32.47"E

*Abbreviations*: *HP* Huangpu District, *HK* Hongkou District, *SJ* Songjiang District

### Study participants

With informed consent, 45 male and female volunteers between 31–59 years-old were recruited and trained for participation (Table 2). The study was carried out in areas without reported local dengue or Zika cases. Human landing catches were conducted where there were no *Aedes*-vectored disease cases during the study period.

**Table 2:** Demographics and blood groups of volunteer participants as human-baits for HDN and HLC

Field monitoring site	Human-bait A			Human-bait B		
	Gender	Blood type (ABO)	Age	Gender	Blood type (ABO)	Age
Site 1	M	O	58	M	B	60
Site 2	F	B	60	M	A	35
Site 3	M	A	31	M	AB	58
Site 4	M	O	57	M	A	47
Site 5	M	O	56	M	AB	55
Site 6	F	O	47	F	A	33
Site 7	M	B	36	M	AB	57
Site 8	M	A	35	M	O	58
Site 9	F	B	26	F	A	57
Site 10	F	AB	59	F	B	59
Site 11	M	O	34	F	B	49
Site 12	F	B	40	F	B	47
Site 13	M	A	59	F	O	45
Site 14	M	A	58	F	O	38
Site 15	M	A	33	M	O	60

*Abbreviations*: *F* female, *M* male

### Mosquito sampling

**Fig. 2:**
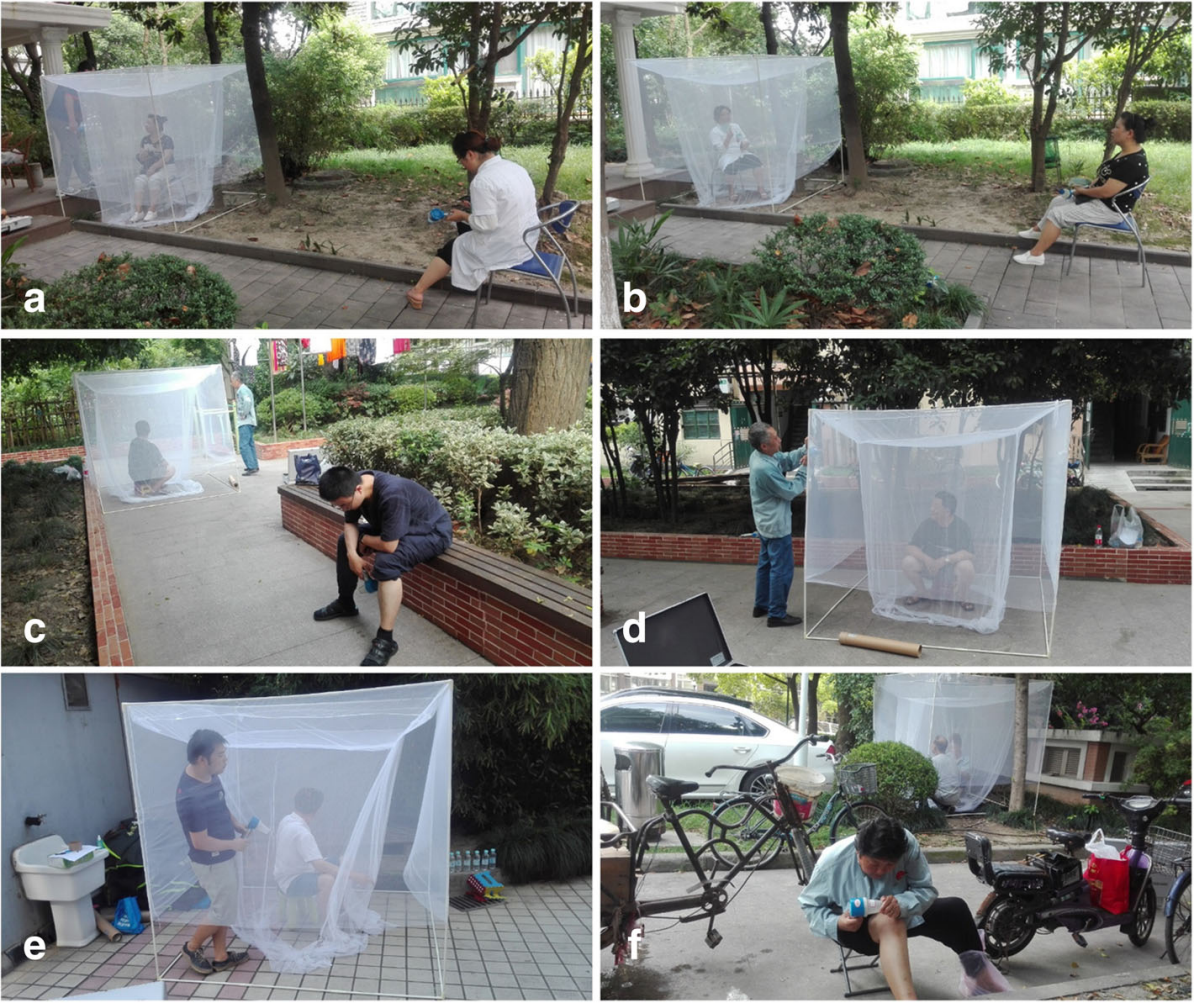
Field mosquito monitoring with HDN and HLC. **a**, **b** The two human baits of HLC and HDN exchanged between the first and the second 30 min to minimize the possible attractant bias. **c**, **f** Participants of HLC attracted mosquitoes with the left or right leg exposed. **d**, **e** The collectors of HDN approach the trap every 5 min to catch mosquitoes resting in front of the outer net and between the nets using a portable battery-powered aspirator

**Fig. 3:**
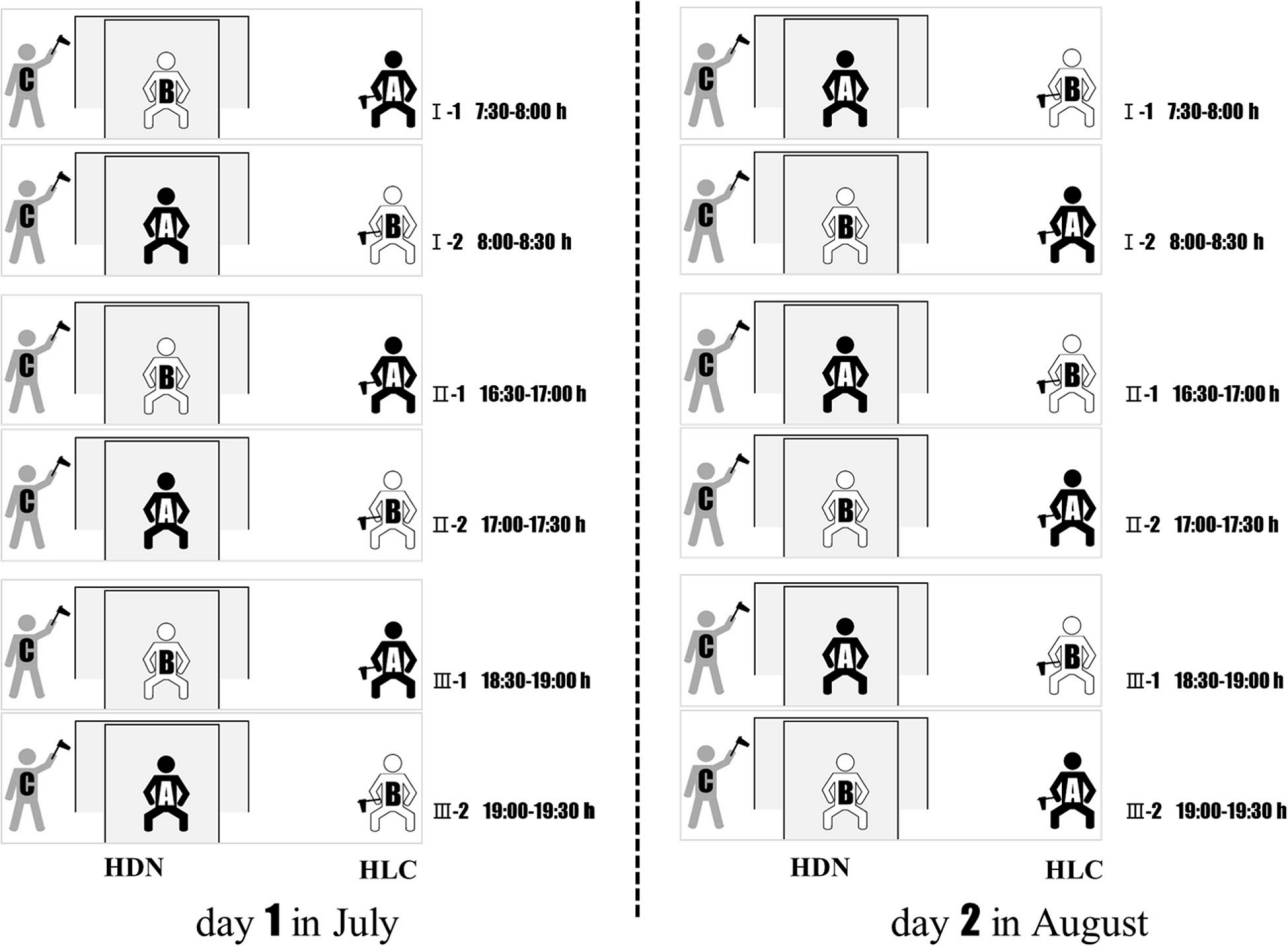
Comparison between HDN and HLC field mosquitoes monitoring at each site (total = 15 sites)

Outdoor HLC and HDN catches were conducted simultaneously at 15 field sites on a sunny day in mid-July. The catches were performed 3 times a day, and each time with a duration of 1 h. These were the early morning (7:30–8:30 h), the pre-sunset late afternoon (16:30–17:30 h) and the post-sunset hours (18:30–19:30 h). The catch comparisons were repeated in mid-August (i.e. *n* = 15 sites × 3 h × 2 d = 90 comparisons for HDN *vs* HLC). At each site, HLC and HDN were positioned 10 m apart (Fig. 2). The 45 participants were randomly divided into 15 groups for the 15 sites, and each group had 3 participants (human bait A, human bait B and collector C) (Table 2, Fig. 3). During the first 30 min, A acted as the attractant and collector for HLC catches, B acted as the attractant and C as the collector for HDN catches. Then A and B exchanged roles during the second 30 min to minimize the possible attractant bias between A and B. Collector C always act as the HDN collector. To eliminate the time bias between the two 30 min sampling periods, the August scheme was modified according to the July results (Fig. 3). Human landing catches were performed by B for the first 30 min and A for the second 30 min. Details of the plan are presented in Fig. 3.

Mosquito samples were taken to the laboratory, killed by freezing, and then counted and identified using taxonomic keys [12].

### Human-baited double net trap

Human-baited double net traps in this study were made of two box nets (inner net size: 140 cm long × 140 cm wide × 210 cm high; outer net size: 200 cm long × 200 cm wide × 170 cm high, net mesh size 1.5 mm), supported by a cube plastic frame (size: 200 cm long × 200 cm wide × 200 cm high), the bottom of the outer net was raised 30 cm above the ground (Fig. 2). The nets were not treated with any insecticide. Human-baited double net trap catches were performed by two participants including one human-bait and one collector. The human-bait (A or B in Fig. 3) rested inside the inner net with two legs exposed, and the collector (C in Fig. 3) with protective clothes and repellent application would approach the trap every 5 min to catch mosquitoes resting in front of the outer net and between the nets using a portable battery-powered aspirator (Fig. 2). For mosquitoes trapped between the nets, collectors bent over and got through the bottom gap of the outer net to perform collection.

### Human landing catch

Human landing catch was performed by a single participant (A or B in Fig. 3), who acted as both attractant and collector. The participants rested about 10 m apart from HDNs with the left or right leg exposed, and collected mosquitoes landing on their exposed legs using a portable battery-powered aspirator (Fig. 2).

### Statistical methods

Data were analyzed using SPSS version 11.5 (SPSS, Inc., Chicago, IL, USA) statistical package. The variance between percentages or proportions (%) were compared by Pearson’s Chi-square test. For quantitative data such as mosquito catch size or mosquito density, most were not normally distributed. After logarithmic transformation of the data, an independent t-test or one-way analysis of variation (one-way ANOVA) was used for analysis. Pearson correlation analysis was used for spatial or temporal sampling yields between HDNs and HLCs. A value of *P* < 0.05 represented a significant difference.

## Results

### Comparison between HLC and HDN for mosquito spatial distribution

A total of 1361 and 574 adult mosquitoes were captured at the 15 sampling sites by HLC and HDN, respectively. The two methods yielded two mosquito species and the individuals collected were predominantly *Ae. albopictus* (HDN: *n* = 1325, 97.35% of total; HLC: *n* = 531, 92.51% of total). The HDN sampling efficiency for *Ae. albopictus* was about 0.40 times that of HLC, and only 79 *Culex pipiens* complex (mainly forms *quinquefasciatus* and *pallens*) were collected (HDN: *n* = 36, 2.65% of total; HLC: *n* = 43, 7.49% of total). Among the adult mosquitoes sampled, females were captured significantly more often than males in both HDN and HLC (Table 3). No difference in the proportion of females was observed between HDN and HLC (80.60 *vs* 82.49%, *χ*^2^ = 0.913, *df* = 1, *P* = 0.339) (Table 3).

**Table 3:** Species and sex composition of adult mosquitoes collected using HDN traps and HLC

Collection methods	*Ae. albopictus*		*Cx. pipiens* complex	
	Female *n* (%)	Male *n* (%)	Female *n* (%)	Male *n* (%)
HLC	1093 (82.49)^*^	232 (17.51)	27 (75.00)^*^	9 (25.00)
HDN	428 (80.60)^*^	103 (19.40)	42 (97.67)^*^	1 (2.23)
Sum	1521 (81.95)^*^	335 (18.05)	69 (87.34)^*^	10 (12.66)

^*^Pearson *χ*^2^ test compared with males, female proportion is significantly higher; *P*-value < 0.05

**Fig. 4:**
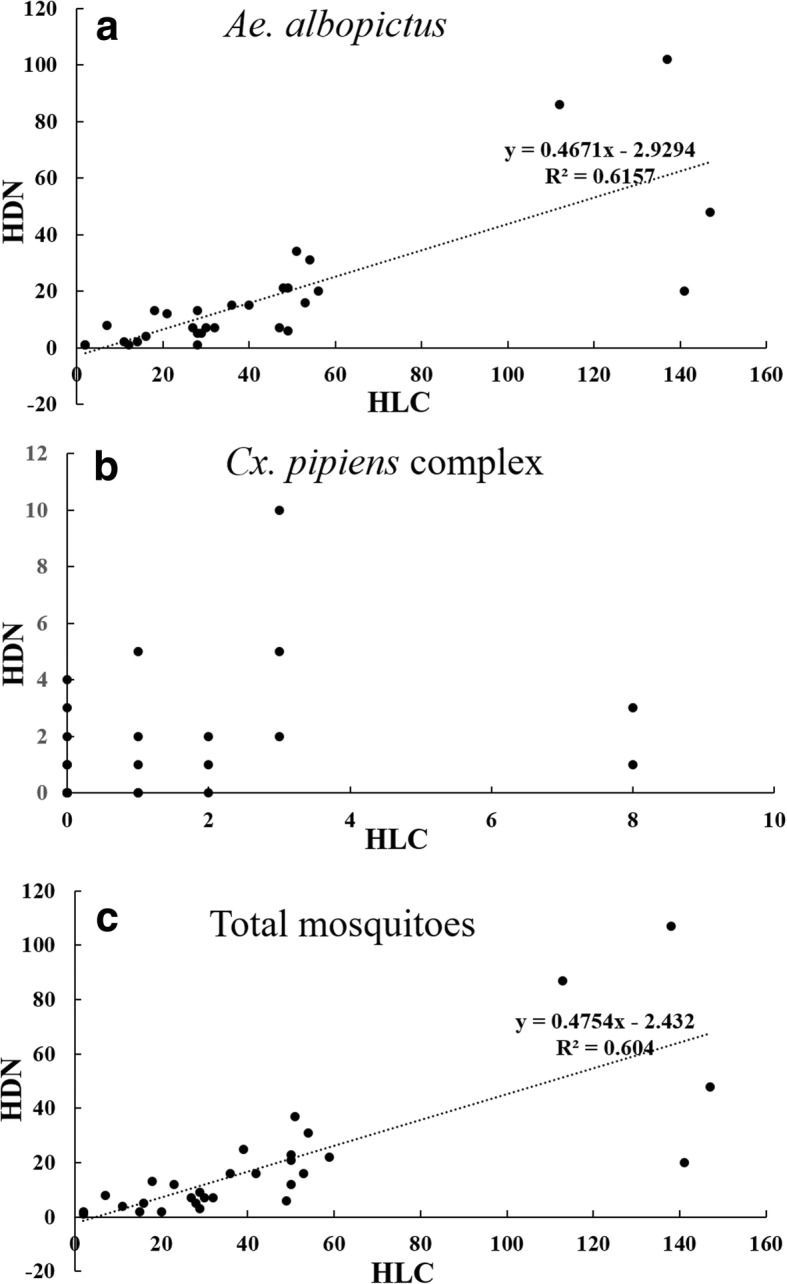
Spatial correlation between HDN and HLC for the number of mosquitoes collected. **a** *Ae. albopictus*. **b** Species of the *Cx. pipiens* complex. **c** Total mosquitoes

Human-baited double net trap yielded *Ae. albopictus* at different densities per hour for the 15 sites, ranging from 0.33 to 31.33 per h. The *Ae. albopictus* density for HLC ranged from 0.67 to 48.00 per h (Table 4). Although the mean *Aedes* catch per h by HLC was significantly higher than HDN (14.72 *vs* 5.90 per h, *t*_(178)_ = 3.151, *P* = 0.003), there was a significantly positive spatial correlation between HLC and HDN for *Ae. albopictus* and the overall number of mosquitoes collected (*Ae. albopictus*: *r*_(90)_ = 0.785, *P* < 0.001; overall: *r*_(90)_ = 0.785, *P* < 0.001) (Fig. 4).

**Table 4:** Mosquito population structure and density at the 15 sites for HDN and HLC

Collection site	HLC				HDN			
	*Ae. albopictus*		*Cx. pipiens* complex		*Ae. albopictus*		*Cx. pipiens* complex	
	Total catch	Mean catch/h	Total catch	Mean catch/h	Total catch	Mean catch/h	Total catch	Mean catch/h
Site 1	43	7.17	0	0.00	11	1.83	1	0.17
Site 2	85	14.17	3	0.50	21	3.50	10	1.67
Site 3	76	12.67	3	0.50	12	2.00	9	1.50
Site 4	69	11.50	0	0.00	47	7.83	3	0.50
Site 5	35	5.83	0	0.00	13	2.17	0	0.00
Site 6	83	13.83	0	0.00	23	3.83	0	0.00
Site 7	4	0.67	0	0.00	2	0.33	1	0.17
Site 8	23	3.83	8	1.33	3	0.50	3	0.50
Site 9	82	13.67	1	0.17	32	5.33	2	0.33
Site 10	288	48.00	0	0.00	68	11.33	0	0.00
Site 11	49	8.17	10	1.67	25	4.17	3	0.50
Site 12	249	41.50	2	0.33	188	31.33	6	1.00
Site 13	97	16.17	3	0.50	42	7.00	2	0.33
Site 14	96	16.00	5	0.83	35	5.83	3	0.50
Site 15	46	7.67	1	0.17	9	1.50	0	0.00
HP (Sites 1–10)	788	13.13	15	0.25	232	3.87	29	0.48
HK (Sites 11–13)	395	21.94	15	0.83	255	14.17	11	0.61
SJ (Sites 14–15)	142	11.83	6	0.50	44	3.67	3	0.25
Total	1325	14.72	36	0.40	531	5.90	43	0.48

No significant difference was observed between HLC and HDN in mean *Culex* catch (0.40 *vs* 0.48 per h, *t*_(178)_ = -0.416, *P* = 0.679), and no significant correlation was found between the two methods of *Culex* collection (Fig. 4).

### Adult mosquito population structure

**Fig. 5:**
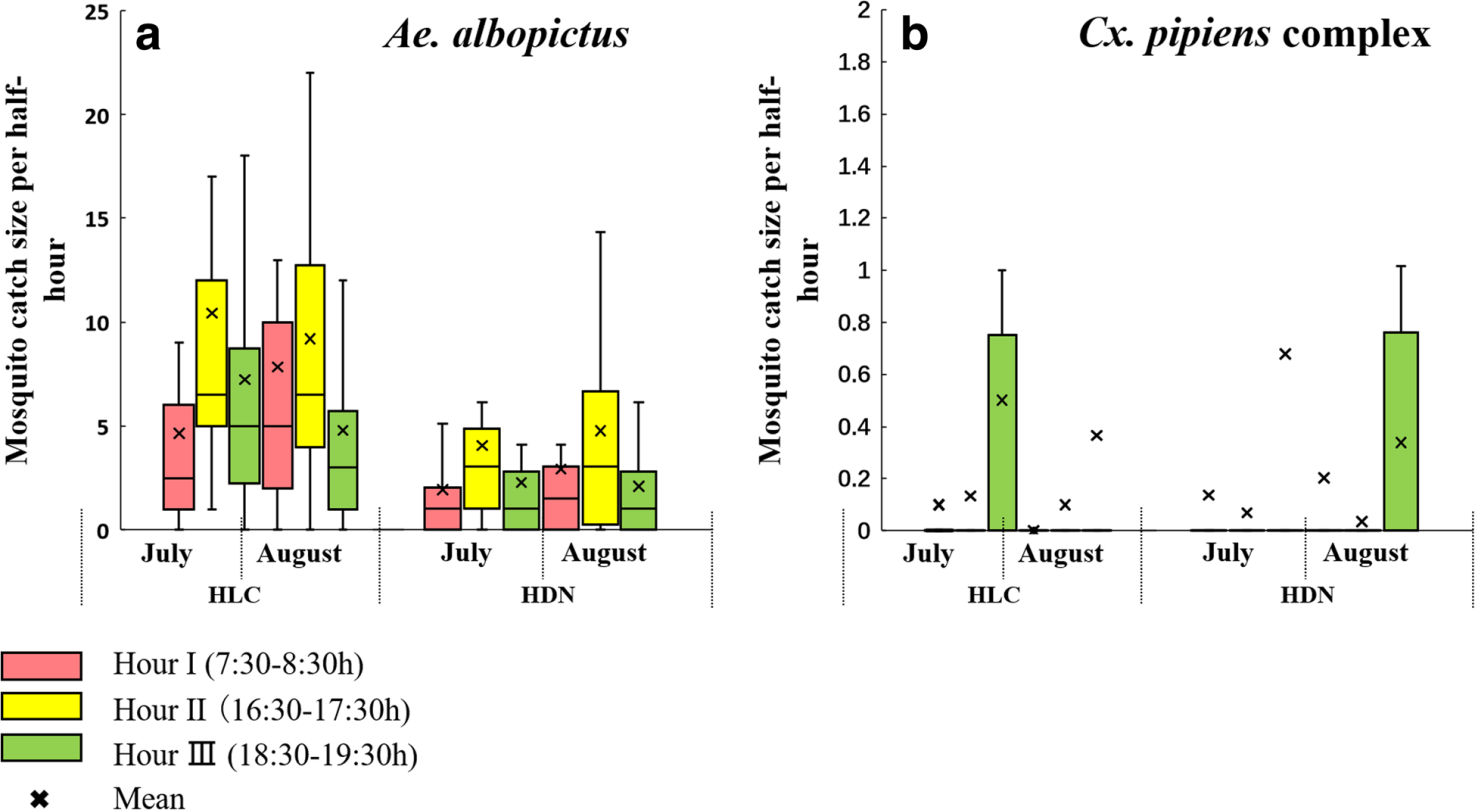
Sampling yields of HLC and HDN in different half-hour blocks. **a** *Ae. albopictus*. **b** Species of the *Cx. pipiens* complex

Due to relatively small sample size, no statistically significant variation of *Ae. albopictus* catches was observed among the different hours of the day, tested by one-way ANOVA for both HDN and HLC (HDN: *F*_(2, 87)_ = 2.359, *P* = 0.101; HLC: *F*_(2, 87)_ = 2.390, *P* = 0.098); but it was apparent that *Ae. albopictus* density at 16:30–17:30 h was higher than at 7:30–8:30 h and at 18:30–19:30 h for both HDN and HLC (HDN: 8.63 *vs* 4.77, 4.30 per h; HLC: 19.63 *vs* 12.50, 12.03 per h) (Table 5, Fig. 5). There was a significantly positive temporal correlation between HLC and HDN of *Ae. albopictus* sampling at different hr (*Ae. albopictus*: *r*_(90)_ = 0.785, *P* < 0.001; overall: *r*_(90)_ = 0.785, *P* < 0.001). The two methods both showed that *Ae. albopictus* were most active during the hour before sunset and less active after sunset.

**Table 5:** Temporal comparison of population structure and density between HDN and HLC

	Time of day	HLC		HDN		*t-*value	*P*-value
		Total catch	Mean catch/h (95% CI)	Total catch	Mean catch/h (95% CI)		
*Ae. albopictus*	Hour I (7:30–8:30)	375	12.50 (8.27–16.73)	143	4.77 (2.59–6.94)	3.256	0.002
	Hour II (16:30–17:30)	589	19.63 (14.04–25.22)	259	8.63 (5.73–11.54)	3.494	0.001
	Hour III (18:30–19:30)	361	12.03 (8.57–15.50)	129	4.30 (2.51–6.08)	3.970	<0.001
	Total	1325	14.72 (12.12–17.33)	531	5.90 (4.55–7.25)	5.929	<0.001
*Cx. pipiens* complex	Hour I (7:30–8:30)	3	0.10 (-0.01–0.21)	10	0.33 (0.08–0.59)	-1.675	0.098
	Hour II (16:30–17:30)	7	0.23 (0.04–0.43)	3	0.10 (-0.01–0.21)	1.194	0.235
	Hour III (18:30–19:30)	26	0.87 (0.34–1.39)	30	1.00 (0.28–1.72)	-0.299	0.765
	Total	36	0.40 (0.21–0.59)	43	0.48 (0.22–0.74)	-0.475	0.635

In this study, most *Cx. pipiens* complex were captured after sunset (Table 5, Fig. 5).

### Half-hour bias and human-bait attraction bias

**Fig. 6:**
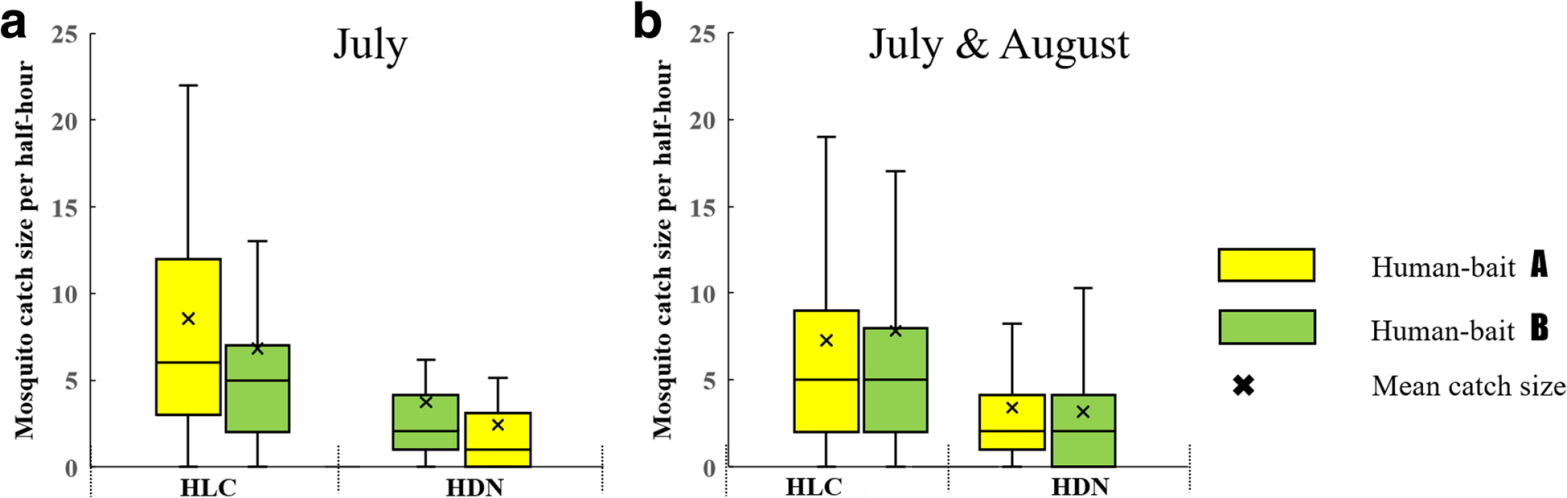
Half-hour (30 min) bias in July. **a** In July, human-bait A performs HLC and HDN catches in the first and the second 30 min, respectively; human-bait B performs HDN and HLC catches in the first and second 30 min, respectively; mosquito yields of the first 30 min were always more than the second 30 min. **b** After scheme modification in August, yields of bait A and B were equivalent overall

In July, human-bait A attracted more *Ae. albopictus* than bait B for HLC (8.56 *vs* 6.82 per 30 min, *t*_(88)_ = 0.862, *P* = 0.391), and human bait A attracted fewer *Ae. albopictus* than bait B for HDN (3.62 *vs* 2.36 per 30 min, *t*_(88)_ = 1.226, *P* = 0.224) (Fig. 6). This means that *Ae. albopictus* collection by HLC and HDN in the first 30 min were significantly greater than the second 30 min. Because of this, we modified the collection plan in August (Fig. 3). Then human-bait A attracted as many mosquitoes as bait B for HLC (7.28 *vs* 7.84 per 30 min, *t*_(178)_ = -0.429, *P* = 0.668) and HDN (3.31 *vs* 3.06 per 30 min, *t*_(178)_ = 0.349, *P* = 0.727) overall (Fig. 6).

**Fig. 7:**
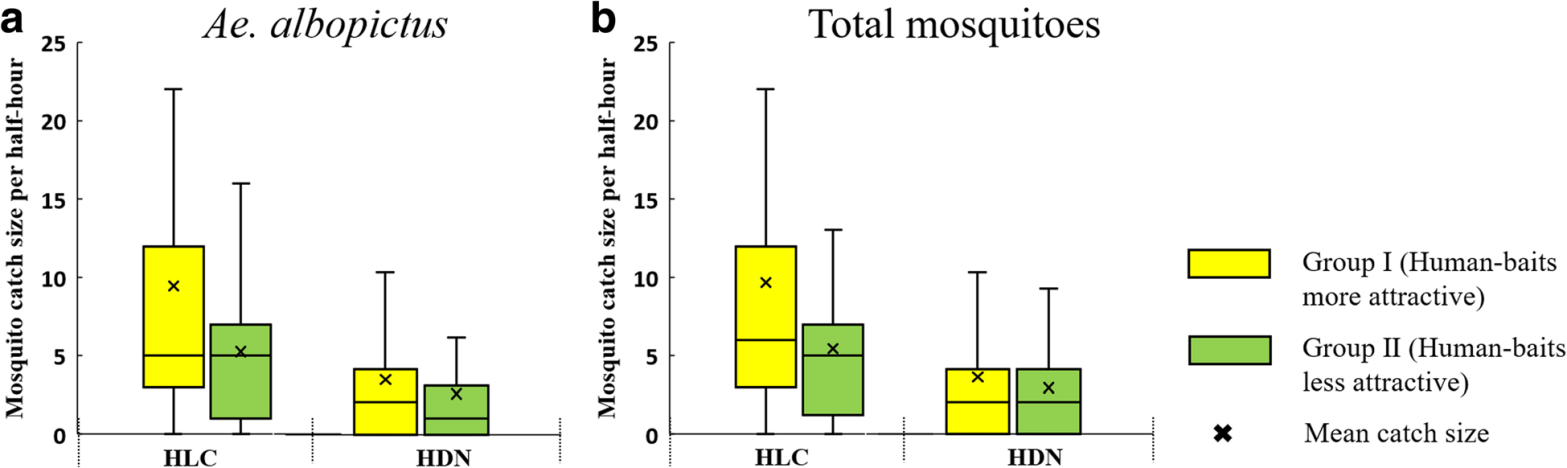
Human-bait attraction bias of HLC. **a** *Ae. albopictus*. **b** Total mosquitoes

When performing HLC catches, we found that human-bait A and bait B in each particular location always had different sampling yields, which may be attributed to the different attraction between A and B (i.e. human-bait attraction bias). We identified 15 human baits with more mosquito yields to form a group of more attractive baits (Group I), and the other 15 formed a group of less attractive baits (Group II). The HLC catch size of Group I was significantly larger than that of Group II (*Ae. albopictus*: 9.47 *vs* 5.26 per 30 min, *t*_(88)_ = 3.274, *P* < 0.001; overall mosquitoes: 9.68 *vs* 5.44 per 30 min, *t*_(88)_ = 3.301, *P* < 0.001). No significant differences were observed for HDN catch size between Group I and Group II (*Ae. albopictus*: 3.38 *vs* 2.51 per 30 min, *t*_(88)_ = 1.283, *P* = 0.201; overall mosquito: 3.52 *vs* 2.86 per 30 min, *t*_(88)_ = 0.954, *P* = 0.341) (Fig. 7). These results suggest that the human-bait attraction bias of HDN catches is not so apparent as for HLC catches.

## Discussion

To our knowledge, this study is the first description of HDN traps used in areas dominated by *Ae. albopictus*. After a field comparison, we found that HDN can be a safer alternative to HLC for *Ae. albopictus* monitoring in Shanghai. Although the *Ae. albopictus* sampling efficiency of HDN was less than that of HLC, HLC may somewhat overestimate human-biting rates by mosquitoes because the human-baits are relatively more available to host-seeking mosquitoes than that under normal circumstance (i.e. staying undisturbed in one place with legs exposed for 30 min or longer) [13, 14]. Thus, the apparently low sensitivity of HDN may represent less of an underestimate than suggested by HLC. Moreover, there was a significant positive spatio-temporal correlation between these two monitoring methods, and the attraction bias found in HLC can be greatly reduced by using HDN.

Interest in the use of appropriate sampling methods commenced in the early 19th century with the discovery that mosquitoes can transmit pathogens that cause diseases in humans and domestic animals [10]. Human landing catches are considered to be the standard for monitoring host-seeking anthropophagic mosquito populations. Human landing catches estimate the human-biting rate, which is needed to assess the mosquito-borne disease risk [15]. Given the inherent risk of conducting HLCs in areas where mosquitoes transmit harmful or deadly infections to humans, alternative traps with or without attractants have been developed, such as non-attractant traps [16–18], light-trap with or without carbon dioxide (CO_2_) [19, 20], and net traps with or without human- or animal-bait [4, 14, 15]. Due to different mosquito species composition or different monitoring scheme designs like variable sampling duration, the results of these trials can vary substantially. Therefore, no universally satisfactory alternative to HLCs has been developed.

Different trapping techniques usually sample different components of a population, and the choice of sampling methods always depends on the type and quality of information required. We compared adult mosquito monitoring between CO_2_-light traps and HLCs in a previous study [5]. The light traps attracted similar number of *Culex* mosquitoes as the HLCs, but the *Aedes* catch was significantly lower in the light traps than for HLC. This was not surprising since light traps are usually ineffective for sampling diurnal active mosquito species [10, 21]. For the anthropophilic and day-biting *Ae. albopictus*, human-baited traps may be the most efficient alternative for HLCs. As a consequence, we adopted HDN as an alternative to HLC for *Aedes* sampling.

The HDN method was first described in 1935 by Gater [22], and the original trap consisted of a large net (300 cm long × 210 cm wide × 210 cm high) with a 90-cm-wide flap rolled up to leave two entrances on each longer side. A person enclosed within a smaller protective net slept inside the large net and unrolled the two flaps to collect the entrapped, unfed, hungry mosquitoes in the end. Since mosquitoes tended to escape too easily, this original design has undergone various modifications. Often a single door-like opening was used [23], sometimes one or more sides of the net were partly rolled up and pinned or tucked into place, or a horizontal slit was made [10, 24]. Alternatively, the entire net can be raised a few cm from the ground [15]. The outer net used in this study was raised 30 cm from the ground to give access to hungry mosquitoes.

Human-baited double net traps have been tested in Africa, Asia and South America with varying outcomes [6–9]. This inconsistency can be attributed to the different mosquito species richness and the trial design. In African areas dominated by anophelines, HLC collected almost four times as many mosquitoes in Nigeria [25] and 7.5 times as many in Cameroon [7] as did the HDN. In western Venezuela [6], HDN trapped only three anophelines compared with 1237 collections using the HLC for 36 h at night. Rubio-Palis & Curtis [6] and Le Goff et al. [7] discarded HDN traps because of their poor efficiency in the collection of anophelines compared with HLCs. For *Aedes* species, the trial in Lao PDR by Tangenaet al. [15] showed that HDN trapped similar numbers of *Anopheles* and *Culex* mosquitoes as HLC and about 0.47 times as many as HLC for *Ae. albopictus*. Mosquito species richness in Shanghai is limited compared with the above-mentioned regions, and in this study, HDN trapped about 0.42 times as many *Ae. albopictus* as HLC. This confirmed that HDN traps are less effective for *Ae. albopictus* sampling compared with HLCs. However, HLC in this study may overestimate *Ae. albopictus* biting rates because: (i) ordinarily, it is unlikely that a human subject would remain still or undisturbed in one place with the skin on their legs exposed for an hour or longer [14]; and (ii) an unusually high biting rate may be encountered at the beginning (the first hour) of any catch [10, 26–28]. Therefore, the relatively lower efficiency of HDN for *Aedes* sampling in this trial might be acceptable.

It was mentioned in other HDN tests that human-baits get out of the inner net and perform collection of mosquitoes trapped between the nets at the end of each hour [15]. Such behaviour could put the human-baits under potential biting-risk when performing mosquito collection without protection, even if the collection time is short. This drawback of HDN performed with one collector, couples with the general weaknesses of HDN that mosquitoes attracted by the human-bait may not always find ways of entering the gap between the two nets, and would not be collected; and that trapped mosquitoes may also escape during the hour before collection. We therefore made some improvements in this test, including: (i) the labor division of bait and collector; (ii) collection of mosquitoes resting both in front of the outer net and between the nets; (iii) collections performed every 5 min. Although labor-intensive, half an hour’s monitoring duration is acceptable. Since peak biting activity periods of *Ae. albopictus* (normally a day-biting species) are relatively well known, landing catches and net trap catches in this study were not conducted with 24-h or 12-h continuous sampling. We chose 3 one-h periods to represent early morning, the pre-sunset and post-sunset periods of the day. The midday period, with direct sunlight and high temperature, was not used. Performing 1-h or 30-min catches is also the local routine mosquito monitoring scheme for emergency when there are imported or local *Aedes*-borne disease cases like dengue or Zika. Corbet & Smith [29] also concluded that it was unnecessary to catch *Aedes* over its entire biting cycle. Only a portion of this time was needed to obtain reliable measurements of density. Sampling duration restricted to 1–2 h around the peak biting time can improve sampling efficiency and reduce the risk of pathogen transmission. This study confirmed that the activity of *Ae. albopictus* in Shanghai peaked in the pre-sunset hour and dropped significantly in the post-sunset hour. Mosquitoes of the *Culex pipiens* complex preferred to bite at night [12], so it was unsurprising that few *Culex* mosquitoes were caught in this study, with density apparently rising in the post-sunset hour during this study.

Mosquito attraction to hosts is mediated by intrinsic, genetic factors as well as extrinsic factors, including heat, water vapor, CO_2_ and various odors emanating from hosts [10]. Since these factors vary among different individuals, different human baits have different levels of mosquito attraction, and human-baited catches can produce a degree of sampling bias. In this study, the HLC catch size of Group I (bait group of more attractive humans) was significantly larger than Group II (bait group of less attractive humans), while no significant variation was observed in the HDN catch size between the two groups. This suggests that the human-bait attraction bias of HDN catches is not as great as in HLC catches. The bias reduction of HDN may be attributed to the trap design of two box nets, which narrowed individual variations by limiting excessive attractive emanations from the hosts inside the inner net. This is also likely to be the reason for the moderate efficiency of HDN.

Compared with light traps or other chemical-attractant traps, HDN is still a labor-intensive sampling method. The trap operation in this study required two participants acting as bait and catcher, and the field preparation of the trap (i.e. frame building) before sampling was more tedious compared to the HLC. The HDN trap design could be improved to make it more portable and easier for field operation. The color of the net tested in this study was white, which makes the black *Ae. albopictus* easier to find. However, other fabric colors and different mesh sizes should be tested.

## Conclusions

With moderate sampling efficiency, significantly positive spatial correlation with HLC, and less human-bait attraction bias, HDN could be a safer alternative to HLC for *Ae. albopictus* monitoring in Shanghai. Peaking in the pre-sunset hour, *Ae. albopictus* catches of HDN should be performed in the hours before darkness. The trap design should be improved to make it more portable and easier to use in the field.

## Abbreviations

ANOVA: Analysis of variance
CO_2_: Carbon dioxide
HDN: Human-baited double net trap
HLC: Human landing catch
